# Fasting increases 18:2-containing phosphatidylcholines to complement the decrease in 22:6-containing phosphatidylcholines in mouse skeletal muscle

**DOI:** 10.1371/journal.pone.0255178

**Published:** 2021-07-26

**Authors:** Nanami Senoo, Takumi Akahori, Hiyori Ichida, Noriyuki Miyoshi, Akihito Morita, Takao Shimizu, Hideo Shindou, Shinji Miura

**Affiliations:** 1 Laboratory of Nutritional Biochemistry, Graduate School of Nutritional and Environmental Sciences, University of Shizuoka, Suruga-ku, Shizuoka, Japan; 2 Laboratory of Biochemistry, Graduate School of Nutritional and Environmental Sciences, University of Shizuoka, Suruga-ku, Shizuoka, Japan; 3 Department of Lipid Signaling, National Center for Global Health and Medicine, Shinjuku-ku, Tokyo, Japan; 4 Department of Lipid Science, Graduate School of Medicine, The University of Tokyo, Bunkyo-ku, Tokyo, Japan; University of Waterloo, CANADA

## Abstract

Fasting stimulates catabolic reactions in skeletal muscle to survive nutrient deprivation. Cellular phospholipids have large structural diversity due to various polar-heads and acyl-chains that affect many cellular functions. Skeletal muscle phospholipid profiles have been suggested to be associated with muscle adaptations to nutritional and environmental status. However, the effect of fasting on skeletal muscle phospholipid profiles remains unknown. Here, we analyzed phospholipids using liquid chromatography mass spectrometry. We determined that fasting resulted in a decrease in 22:6-containing phosphatidylcholines (PCs) (22:6-PCs) and an increase in 18:2-containing PCs (18:2-PCs). The fasting-induced increase in 18:2-PCs was sufficient to complement 22:6-PCs loss, resulting in the maintenance of the total amount of polyunsaturated fatty acid (PUFA)-containing PCs. Similar phospholipid alterations occurred in insulin-deficient mice, which indicate that these observed phospholipid perturbations were characteristic of catabolic skeletal muscle. In lysophosphatidic acid acyltransferase 3-knockout muscles that mostly lack 22:6-PCs, other PUFA-containing PCs, mainly 18:2-PCs, accumulated. This suggests a compensatory mechanism for skeletal muscles to maintain PUFA-containing PCs.

## Introduction

Skeletal muscle adapts its metabolism to suit nutritional status and environmental conditions. Fasting stimulates muscle catabolic processes to survive nutrient deprivation. Breakdown of structural muscle proteins supplies amino acids for gluconeogenesis and ATP production, resulting in the loss of muscle mass [1, 2]. Lipolysis using circulating lipoprotein-triacylglycerol (TG) and intramuscular TG provides free fatty acids for β-oxidation in skeletal muscle [1, 3, 4]. Many nutritional signals, including hormones, glucose, amino acids, and growth factors, are involved in fasting-induced skeletal muscle adaptation [5]. For example, plasma insulin concentration is low during fasting, which suppresses translation and activates protein degradation processes, such as ubiquitin/proteasome system and autophagy [6, 7]. Insulin is a regulator of lipolysis in skeletal muscle. Insulin infusion in human subjects suppresses the activity of lipoprotein lipase (LPL), which hydrolyzes circulating lipoprotein-TGs [8]. In mice, LPL expression is upregulated due to insulin deficiency [9].

Glycerophospholipids are main components of cellular membrane and possess high structural diversity due to various polar-heads and acyl-chains. It is believed that phospholipid diversity plays crucial roles in modulating cell functions through membrane physical properties and membrane-embedded proteins [10–15]. Acyl-chains that bind to the glycerol-backbone differs in tissues [16, 17], and this also underscores the functional significance of phospholipid diversity within an organism.

To investigate skeletal muscle phospholipid profiles, fatty acids derived from the phospholipid fraction extracted via thin-layer chromatography (TLC) or solid-phase separation were analyzed. These approaches determined that exercise training [18–20] and muscular disease [21] are factors that alter the composition of fatty acids in muscular phospholipids. Recent studies using liquid-chromatography mass spectrometry (MS) (LC-MS) and imaging MS, have enabled more accurate detection of each phospholipid species. They provide a profound insight into skeletal muscle phospholipid alterations under several conditions, including exercise training [22, 23], muscle-wasting phenotype [24], and fiber-type specificity [25]. Nutritional status is another factor that induces phospholipid alteration in skeletal muscle. Long-term feeding of a high-fat diet increases 20:4-containing phospholipids, especially phosphatidylcholine (PC) (18:0/20:4) in rats [22]. In mice fed with high-fat diets, PC and PE containing n-6 polyunsaturated fatty acids (PUFAs), such as 20:4 and 22:5, were increased, while those containing n-3 PUFA were decreased [26]. In addition, dietary fat sources influence skeletal muscle phospholipid profiles [27, 28]. These studies suggest that phospholipid profiles are associated with skeletal muscle metabolism and function. However, the precise mechanisms and physiological significance of skeletal muscle phospholipid alterations remain unclear. Although fasting affects many skeletal muscle metabolic events, its effect on skeletal muscle phospholipid profiles has not been determined.

Here, to reveal if and how fasting affects skeletal muscle phospholipid profiles, we conducted phospholipid analyses using fasted and insulin-deficient mice. We demonstrated that fasting induces decreases in 22:6-containing PCs (22:6-PCs) and increases in 18:2-containing PCs (18:2-PCs). Surprisingly, the fasting-induced 18:2-PCs increase is sufficient to complement the 22:6-PCs decrease, and this compensatory effect maintains the total amount of PUFA-containing PCs. These phospholipid alterations were also observed in insulin-deficient mice. In lysophosphatidic acid acyltransferase (LPAAT) 3-KO muscles that mostly lack 22:6-PCs, other PUFA-containing PCs, especially 18:2-PCs, accumulate. These results altogether suggest a compensatory mechanism by which skeletal muscle maintains the PUFA-containing PCs balance.

## Materials and methods

### Animals

C57BL/6J mice were obtained from Japan SLC, Inc. (Shizuoka, Japan). For the fasting experiments, male 10 week-old mice were randomly assigned to three groups. They were either allowed to feed ad libitum (fed) or subjected to fasting for 24 h or 48 h. Mice were fed a normal diet (MF; CLEA Japan, Tokyo, Japan). LPAAT3-KO mice were generated as previously reported [29]. Male nine-13 week-old LPAAT3-KO mice and wild-type (WT) littermates were used.

Streptozotocin (STZ) (Wako), dissolved in ice-cold 0.05 M citrate buffer (pH 4.5) at a dose of 100 mg/kg body weight was intraperitoneally injected into nine week-old male mice. Control mice received an equal volume of citrate buffer. Three days after STZ injection, mice with high blood glucose concentrations (> 350 mg/dL) were used for experimentation. Skeletal muscles were harvested seven days after STZ injection.

Mice were anesthetized with 4% isoflurane (Isofluran CP®, CP-Pharma Handelsgesellschaft mbH, Burgdorf, Germany) in 100% oxygen in an anesthetic chamber (with sliding cover, Evonik Plexiglas, 240 × 140 × 120 mm), followed by euthanasia by cervical dislocation. The extensor digitorum longus (EDL), soleus, and gastrocnemius muscles were removed and rapidly frozen in liquid nitrogen. Muscle samples were stored at -80°C until processing. Mice were cared for in accordance with the National Institutes of Health Guide for the Care and Use of Laboratory Animals and our institutional guidelines. All animal experiments were conducted with the approval of the Institutional Animal Care and Use Committee of the University of Shizuoka (#165122, 185208).

### Phospholipid analysis

Lipid extraction and quantitative analyses of PC were performed using a triple quadrupole mass spectrometer LCMS-8040 (Shimadzu, Kyoto, Japan) equipped with an electrospray source ionization probe, as described previously [23], with certain modifications. PC (17:0/17:0) (Avanti Polar Lipids, Alabaster, AL) was added to each sample during extraction as an internal standard. For LC analysis, an Accucore RP-MS column (2.6 μm, 2.1 mm × 50 mm, Thermo Fisher Scientific, Waltham, MA) was used. Mobile phase A consisted of water/acetonitrile (60:40, v/v) and mobile phase B consisted of isopropanol/acetonitrile (90:10, v/v). Both mobile phases A and B were supplemented with 10 mM ammonium formate and 0.1% formic acid. The flow rate was 0.35 mL/min. The gradient was as follows: 40% B at 0 min, 40% B at 2 min, 52% B at 8 min, 60% B at 20 min, 100% B at 25 min, and 40% B at 30 min. For MS analysis, we performed multiple reaction monitoring with the transitions [M + NH_4_]^+^ → 184 for PC in the positive ionization mode, focusing on species abundant in mouse skeletal muscle that we previously identified [24]. The relative peak area for each species was normalized to the peak area of the internal standard and muscle weight.

### mRNA analysis

Total RNA from skeletal muscle was isolated using RNAiso Plus (Takara, Shiga, Japan, 9109). cDNA was synthesized using a PrimeScript^TM^ RT reagent Kit (Takara, RR047A). Quantitative RT-PCR was performed using TB Green Premix Ex Taq^TM^ II (Takara, RR820A) and Thermal Cycler Dice® Real Time System (Takara). mRNA expression was normalized to that of 36B4. Mouse-specific primer pairs are shown in S1 Table.

### Statistical analyses

Subsequent to autoscaling, mean-centering, and scaling by standard deviation on a per-peak basis as pretreatment, a principal component analysis (PCA) was conducted using GraphPad Prism ver. 9. Other data were analyzed by student’s t-test or one-way ANOVA. In cases of significant differences, each group was compared to each of the other groups using Tukey’s multiple comparisons test (GraphPad Prism ver. 9 and JMP ver. 11). Values are expressed as the mean ± SE. Two-tailed Pearson’s correlation tests were performed using GraphPad Prism ver. 9.

## Results

### Fasting altered skeletal muscle phospholipid profiles

**Fig 1:**
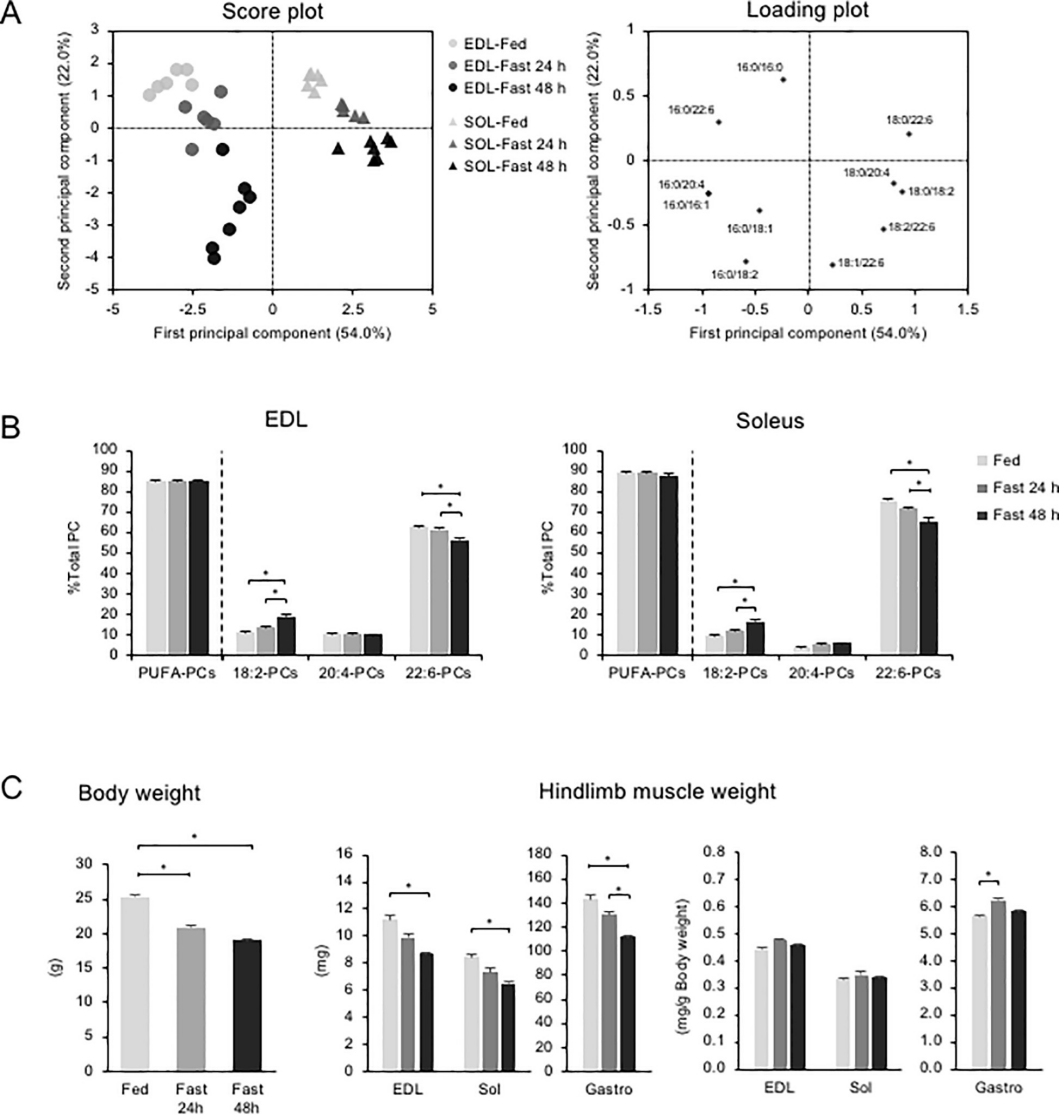
Effects of fasting on skeletal muscle PC profiles. (A) Score plots (left) and loading plots (right) of PCA for measured PC species in EDL and soleus from mice allowed to feed ad libitum (Fed) and those subjected to 24 and 48 h of fasting (Fast). (B) Proportions of each PC group to total PC sorted by acyl-chains at sn-2 position. (C) Body weight and hindlimb muscle weight at dissection. Values represent mean ± SE (n = 5–7); *P < 0.05 (vs. Fed).

As shown in the PCA score plots (Fig 1A), the second principal component (y-axis) scores of EDL and soleus were clearly separated along the y-axis between the fed group and fast group, which indicates that prolonged fasting had a considerable effect on phospholipid profiles. The second principal component positively correlated with PC (16:0/16:0), PC (16:0/22:6), and PC (18:0/22:6), while it had a strong negative correlation with PC (16:0/18:2) and PC (18:1/22:6) (Fig 1A).

To further characterize fasting-induced phospholipid alterations, we sorted PC species by acyl-chains at sn-2 position, identified as previously described [30], and then calculated the proportions of each group to total PC (Fig 1B). 22:6-PCs accounted for the majority of PC in both muscles in fed states. The soleus contained a slightly higher amount of 22:6-PCs than EDL. Fasting decreased the 22:6-PCs proportion, especially PC (16:0/22:6) and increased the 18:2-PCs proportions, such as PC (16:0/18:2) and PC (18:0/18:2) (S1A Fig). Importantly, the sums of PUFA-containing PCs (18:2-, 20:4-, and 22:6-PCs) were invariable between fed and fasted statuses in both the EDL and soleus (Fig 1B). Although fasting increased certain 22:6-PCs, for example, PC (18:1/22:6) and PC (18:2/22:6) in the EDL, the decrease in PC (16:0/22:6) had a larger effect on the 22:6-PCs proportion that counteracted these minor effects (S1A and S1B Fig). These results suggest that the 22:6-PCs decreases during fasting are compensated by 18:2-PCs increases, which might play a role in maintaining PUFA-containing PCs at a consistent level in skeletal muscle. After fasting, body weight and muscle weight significantly decreased (Fig 1C). The normalized muscle weight to body weight ratio was unaltered in the EDL and soleus, but increased in the gastrocnemius at 24 h (Fig 1C).

### Effects of insulin on muscle phospholipid profiles

**Fig 2:**
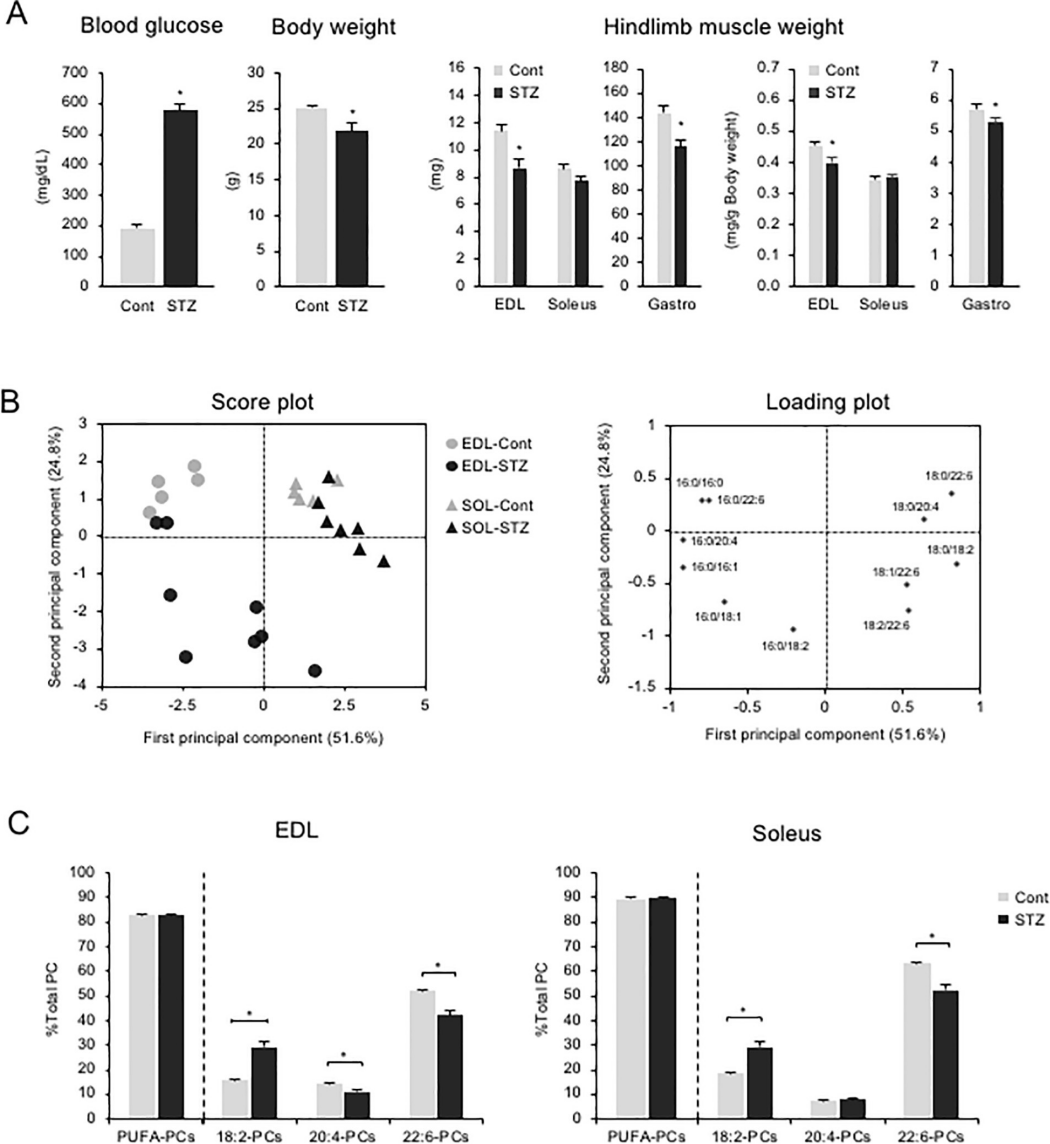
Effects of insulin on skeletal muscle PC profiles. (A) Blood glucose levels, body weight, and hindlimb muscle weights at dissection of control (Cont) and STZ-injected (STZ) mice. (B) Score plots (left) and loading plots (right) of PCA for measured PC species in EDL and soleus from Cont and STZ mice. Proportions of each PC group to total PC sorted by acyl-chains at sn-2 position. Values represent mean ± SE (n = 5–8); *P < 0.05.

Insulin concentration, which changes in response to nutrient states, is closely associated with fasting-induced skeletal muscle metabolism. To assess the involvement of insulin in fasting-induced phospholipid alterations, we injected STZ, which destroys β cells and causes insulin deficiency. STZ-injected mice had high blood glucose levels, in agreement with a previous report [9], and reduced body and hindlimb muscle weights (Fig 2A).

In the PCA score plots, the second principal component (y-axis) scores were divided between control and STZ-injected mice, especially in EDL, which suggests that the second principal component scores were negatively related to insulin-depletion due to STZ injection (Fig 2B). The loading plots showed that PC (16:0/18:2) had the strongest negative correlation with the second principal component, while PC (18:0/22:6), PC (16:0/22:6), and PC (16:0/16:0) had positive correlations (Fig 2B), in agreement with fasting-affected profiles (Fig 1A).

Overall, STZ-injection had similar effects as fasting on EDL and soleus phospholipid profiles (Fig 2C, S2A Fig). STZ-injection rendered the 22:6-PCs proportion reduced and the 18:2-PCs proportion increased, which resulted in no difference in the sum of PUFA-containing PCs between control and STZ-injected mice (Fig 2C). Since both long-term fasting and destruction of pancreatic beta cells by STZ injection cause a reduction in blood insulin levels, insulin concentration might be associated directly or indirectly with fasting-induced phospholipid alteration and may play a role in maintaining the proportion of PUFA-containing PCs.

### Gene expression related to phospholipid synthesis and remodeling

**Fig 3:**
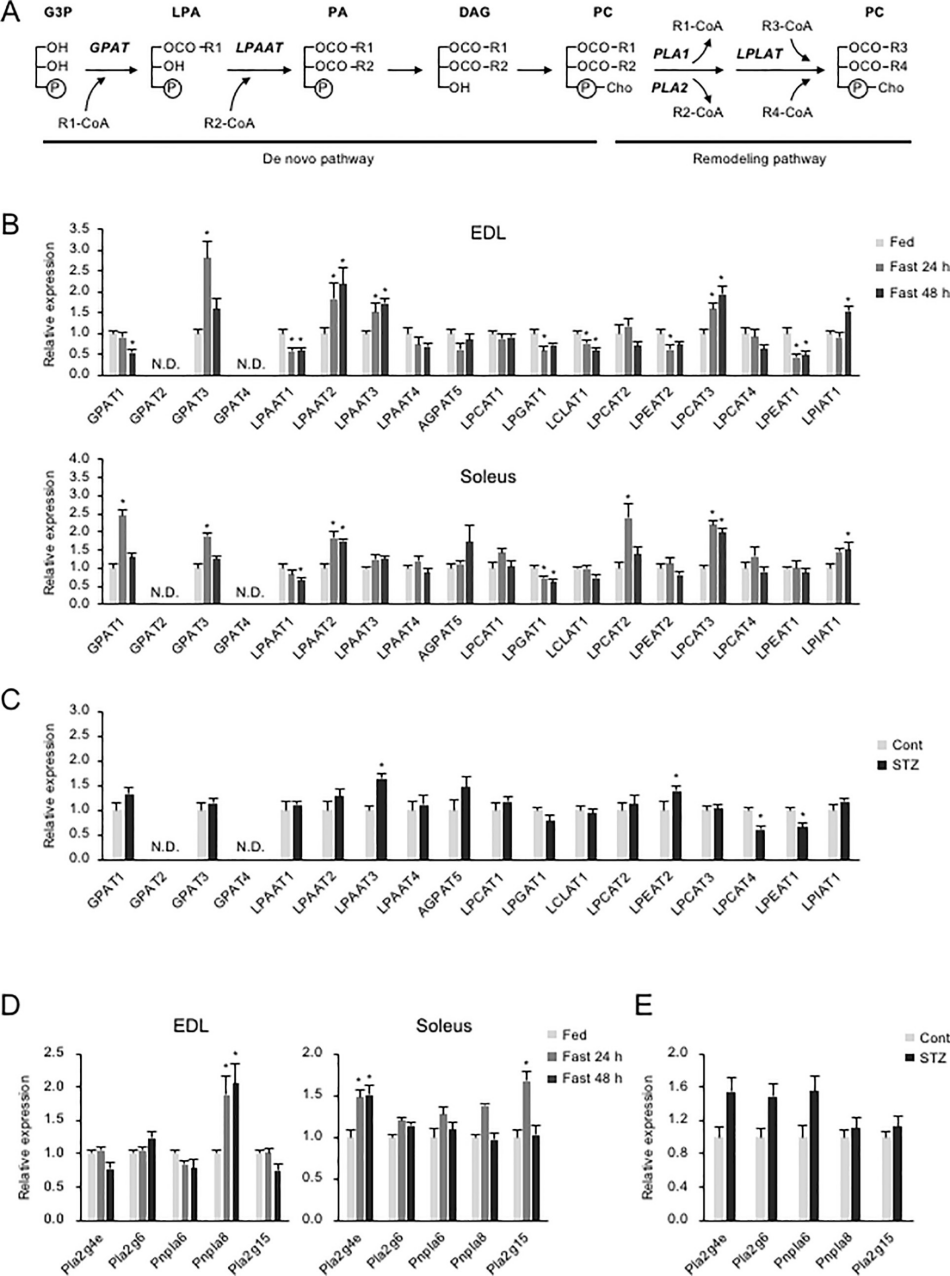
Acyltransferase and PLA_2_ gene expression levels are affected by fasting and insulin. (A) Schematic representation of phospholipid biosynthesis. In the first step of the *de novo* pathway, glycerophosphate acyltransferase (GPAT) incorporates acyl-CoA into glycerol-3-phosphate (G3P) and generates lysophosphatidic acid (LPA). The second acyl-CoA is incorporated into LPA to generate PA by lysophosphatidic acid acyltransferase (LPAAT). Glycerophospholipid fatty acid composition is altered in the remodeling pathway through the concerted action of phospholipase A (PLA) and lysophospholipid acyltransferase (LPLAT). PA, phosphatidic acid; DAG, diacylglycerol; PC, phosphatidylcholine; Cho, choline. (B, D) mRNA expression of acyltransferases in the EDL and soleus (B) and PLA_2_ (D) from ad lib feeding (Fed) and fasting (Fast) mice (n = 6–7). Values represent mean ± SE; *P < 0.05 (vs. Fed). (C, E) mRNA expression of acyltransferases (C) and PLA_2_ (E) in gastrocnemius from control (Cont) and STZ-injected (STZ) mice (n = 5–8). Values represent mean ± SE; *P < 0.05.

Acyltransferases incorporate acyl-CoAs into the phospholipid backbone through *de novo* and remodeling pathways (Fig 3A). Differences in enzyme substrate preferences contribute to the acyl-chain diversity of cellular phospholipids. Reportedly, LPAAT2, lysophosphatidylcholine acyltransferase (LPCAT) 3, and glycerophosphate acyltransferase (GPAT) 3 are responsible for the incorporation of 18:2-CoA [16, 31, 32]. LPCAT3 is also related to 20:4-CoA incorporation [31]. 22:6-CoA is preferentially recognized by LPAAT3, LPAAT4, and lysophosphatidylethanolamine acyltransferase (LPEAT) 2 [16, 33, 34]. To examine whether acyltransferases are associated with fasting-induced phospholipid alteration, we measured their expression. Fasting enhanced the expression of LPAAT2 and LPCAT3 in the EDL and soleus, which might be involved in the 18:2-PCs increase during fasting (Fig 3B). However, the expression levels of both enzymes were not affected by STZ injection (Fig 3C). Among acyltransferases that prefer 22:6-CoA, LPEAT2 expression decreased by fasting in the EDL but not in the soleus. LPAAT3 expression increased by fasting in the EDL, but not altered in the soleus. LPAAT4 expression was not altered (Fig 3B). STZ-injection also increased LPAAT3 expression but did not affect LPAAT4 (Fig 3C). Given the 22:6-PCs decreases seen in both the EDL and soleus, it would be inadequate to explain 22:6-PCs decreases during fasting based on the expression levels of these acyltransferases. Phospholipase A_2_ (PLA_2_) is required for the remodeling at sn-2 position where the majority of PUFAs bind to glycerol backbone of skeletal muscle PC [35]. Among them, Pnpla8 and Pla2g4e were upregulated by fasting in the EDL and soleus, respectively; however, STZ had no effect on expression (Fig 3D and 3E).

### Gene expression related to fatty acid desaturation and elongation

**Fig 4:**
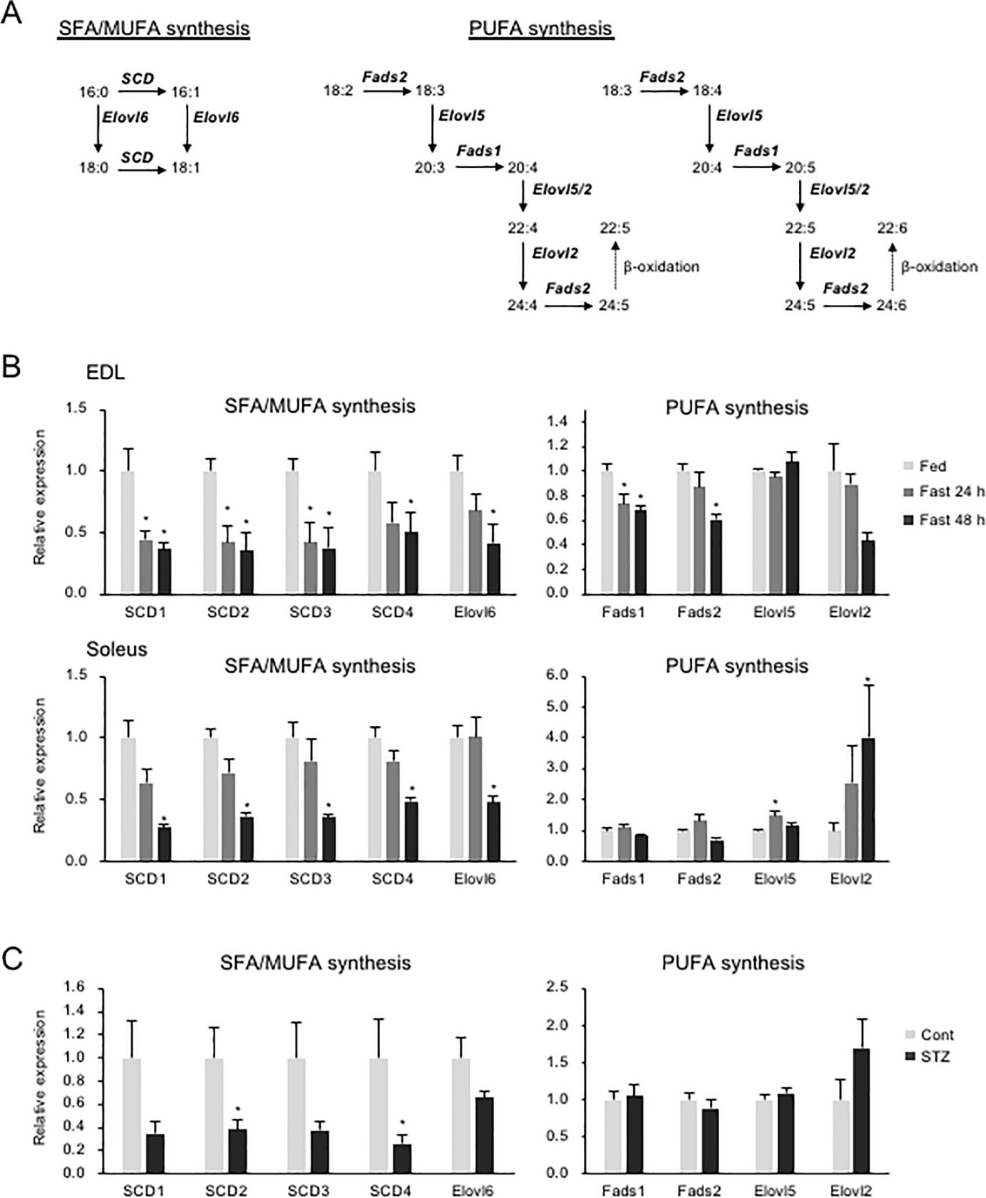
Gene expression levels of desaturases and elongases are affected by fasting and insulin. (A) Schematic representation of fatty acid synthesis. In SFA/MUFA synthesis, SCD and Elovl6 catalyze the addition of double bonds and carbon chain elongation, respectively. 18:2 and 18:3 are converted to the respective n-6 and n-3 PUFA series, ending with peroxisomal beta-oxidation. (B) mRNA expression of desaturases and elongases in EDL and soleus from ad lib feeding (Fed) and fasting (Fast) mice (n = 6–7). Values represent mean ± SE; *P < 0.05 (vs. Fed). (C) mRNA expression of desaturases and elongases in gastrocnemius from control (Cont) and STZ-injected (STZ) mice (n = 5–8). Values represent mean ± SE; *P < 0.05.

Stearoyl-CoA desaturases (SCD) and elongation of very long chain fatty acids protein (Elovl) 6 modulate cellular saturated fatty acids (SFAs) and monounsaturated fatty acids (MUFAs), which originate mainly from glucose-derived acetyl-CoA (Fig 4A) [36]. Fasting reduced the expression of all four mouse SCD isoforms and Elovl6 in the EDL (Fig 4B). Similar changes were observed in the soleus muscle (Fig 4B). The expression of SCD2 and 4 was decreased by STZ-injection (Fig 4C). However, the levels of 16:0-PCs, which would accumulate if SCDs and Elovl6 expressed low, were reduced by both fasting and STZ-injection (S3 Fig). Fatty acid desaturase (Fads) 1 and 2 and Elovl5 and 2 are responsible for PUFA synthesis (Fig 4A) [36]. Fasting reduced the expression of Fads1 and 2 in the EDL; however, this was not consistent with the alterations in the soleus (Fig 4B). STZ had no effect on PUFA synthesis-related genes (Fig 4C).

### Phospholipid profile alterations in LPAAT3-KO muscles

Although the enzymic mechanism remains unclear, our results support the hypothesis that the total amount of PUFA-containing PCs is maintained in skeletal muscle by balancing the proportions of 22:6-PCs and 18:2-PCs. To determine if the loss of 22:6-PCs is a trigger that modulates the amount of 18:2-PCs, we utilized LPAAT3-KO mice that possess near depleted levels of 22:6-PCs in tissues rich in 22:6 [29, 37].

**Fig 5:**
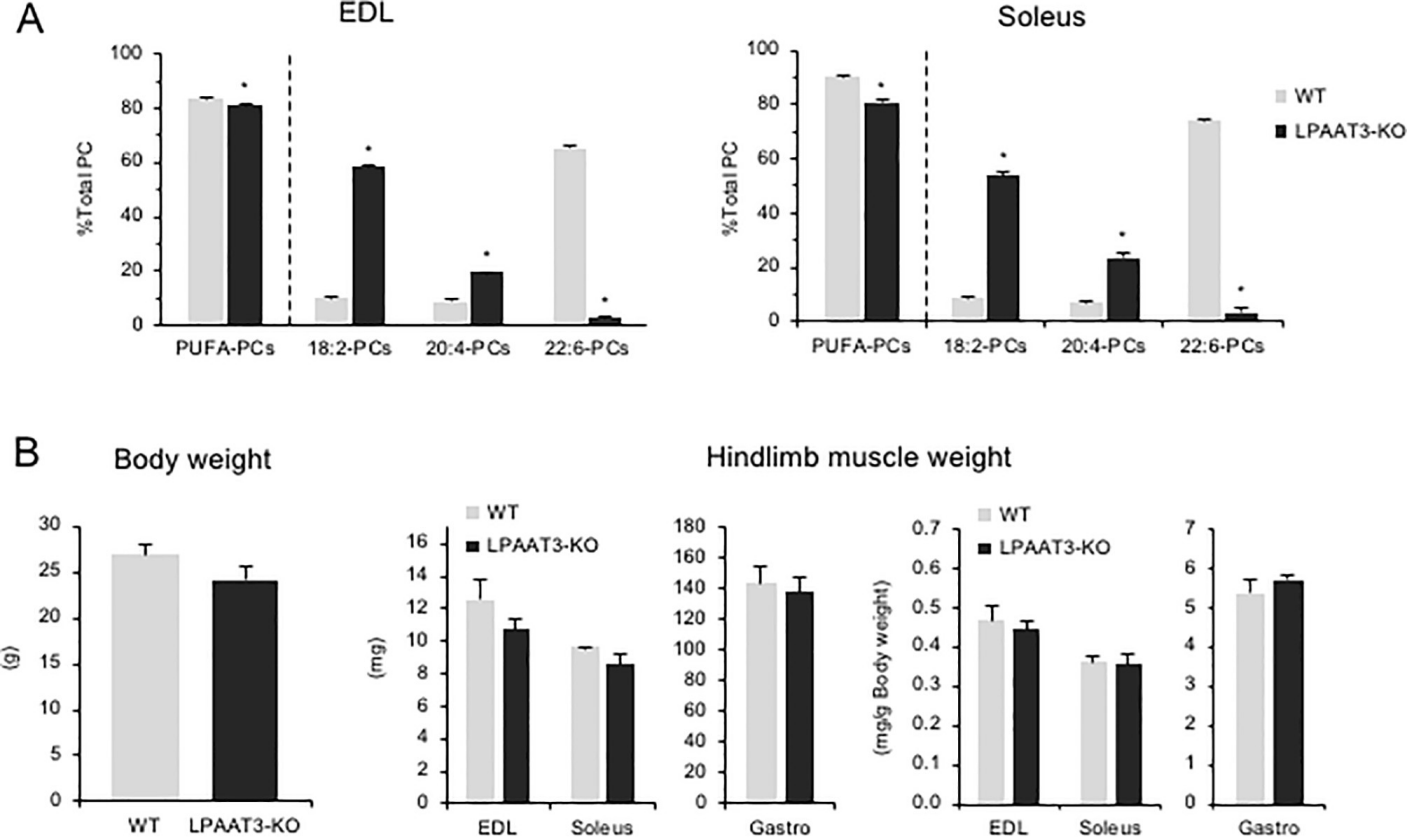
PC profiles in LPAAT3-KO muscles. (A) Proportions of each PC group to total PC sorted by acyl chains at the sn-2 position. (B) Body weight and hindlimb muscle weights at dissection. Values represent mean ± SE (n = 3–4); *P < 0.05.

The levels of all 22:6-PCs species measured were significantly lower in LPAAT3-KO muscles than in WT, but the levels of 18:2-PCs species significantly increased (Fig 5A, S4 Fig). In terms of the proportion of PUFA-containing PCs, the 22:6-PCs decreases in LPAAT3-KO muscles were substantially complemented by a large increase in 18:2-PCs (Fig 5A). 20:4-PCs increased in LPAAT3-KO muscles (Fig 5A), and this may also contribute to the complementation of 22:6-PCs. These results indicate that loss of 22:6-PCs due to LPAAT3 depletion results in compensatory accumulation of other PCs, especially 18:2-PCs, which may contribute to maintaining a certain amount of PUFA-PCs in skeletal muscle. However, we found no difference in body and hindlimb muscle weights between LPAAT3-KO and WT mice (Fig 5B).

### Correlation between phospholipid profiles and muscle mass

**Fig 6:**
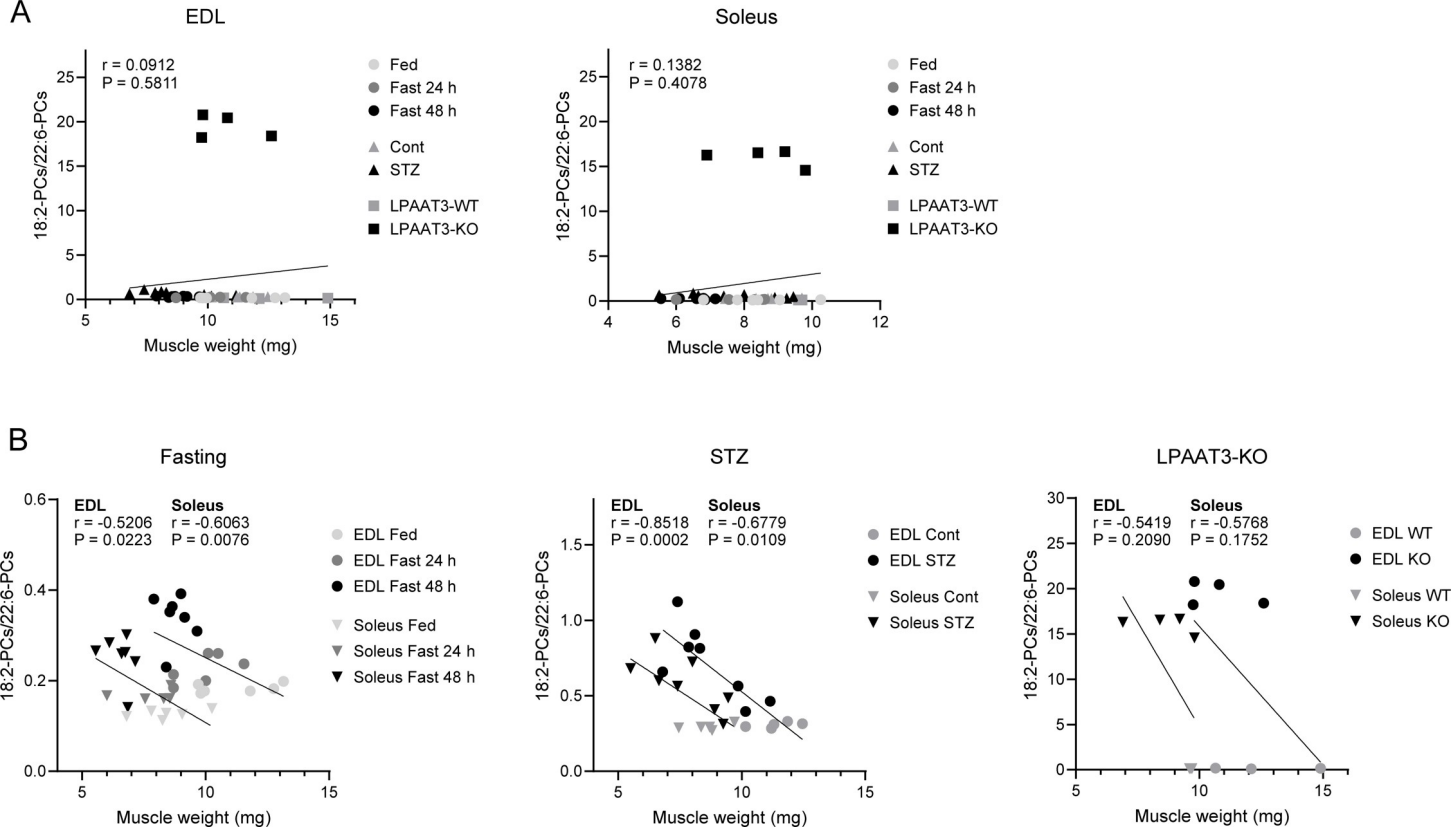
Correlation analyses between PC alteration and muscle mass. Two-tailed Pearson’s correlation tests for EDL and soleus were performed with the ratio of 18:2-PCs/22:6-PCs and muscle weight in all models. These included fasting, ad lib feeding (Fed), and fasting (Fast) mice (n = 6–7); STZ injection, control (Cont) and STZ-injected (STZ) mice (n = 5–8); WT and LPAAT3-KO mice (n = 3–4) (A) and individual models (B).

To estimate the relationship between the alteration of PC profiles and muscle mass, we performed correlation analyses (Fig 6). The tests for the EDL and soleus from all models, fasting, STZ injection, and LPAAT3-KO mice showed no significant correlation between the ratio of 18:2-PCs/22:6-PCs and muscle mass (Fig 6A). Focusing on individual models, fasting and STZ injection yielded negative correlation between 18:2-PCs/22:6-PCs and muscle mass in the EDL and soleus (Fig 6B), suggesting that PC profiles are altered by progressive muscle catabolism. No significant correlation was observed in LPAAT3-KO mice (Fig 6B).

## Discussion

In this study, we determined the effects of prolonged fasting on skeletal muscle phospholipid profiles. In the fed states, 22:6-PCs accounted for the majority of skeletal muscle PCs, followed by 18:2-PCs and 20:4-PCs. Overall, fasting rendered 22:6-PCs decreased but other PUFA-containing PCs increased instead, especially 18:2-PCs (Fig 1). In the insulin-depleted state due to STZ injection, the phospholipid perturbations were consistent with those induced by fasting (Fig 2). Importantly, in both fasting and insulin-depleted statuses, decreases in 22:6-PCs were adequately compensated for by increases in 18:2-PCs (Figs 1 and 2). It seems that a compensatory mechanism functions in skeletal muscle to maintain a certain amount of PUFA-containing PCs. This notion is supported by the PC profiles of LPAAT3-KO muscles, in which loss of 22:6-PCs led to a considerable accumulation of other PCs, especially 18:2-PCs, although total PUFA-containing PC levels were still lower in LPAAT3-KO muscles (Fig 5).

We observed that the expression of acyltransferases that prefer 18:2-CoA, namely LPAAT2 and LPCAT3, was enhanced by fasting; however, no consistent alteration was found upon STZ-injection. In addition, no acyltransferase could adequately explain 22:6-PCs decreases in both fasting and STZ-injected conditions (Figs 1, 2 and 3). PLA_2_ expression was also inconsistent between the EDL and soleus during fasting (Fig 4). These results indicate that modulations of phospholipid acyl-chains via acyltransferases and PLA_2_ are unlikely to be the major determining factors for phospholipid perturbations during fasting. Similarly, PUFA synthesis-related genes did not change consistently between the EDL and soleus, nor between fasting and STZ-injected statuses (Fig 4). SFA/MUFA synthesis-related genes were downregulated by fasting and STZ-injected statuses. This is reasonable, given that sterol regulatory element-binding protein (SREBP)-1c, which is activated by insulin, coordinates many genes involved in fatty acid synthesis, including SCDs and Elovl6 [38, 39]. Theoretically, it is expected that the downregulation of SCDs and Elovl6 would cause 16:0-PCs accumulation. However, 16:0-PCs were reduced in both fasting and STZ-injected groups (S3 Fig), which suggests that the fatty acid modulations by SCDs and Elovl6 during fasting do not cause phospholipid perturbations. Even though we observed a drastic decrease in 22:6-PCs in LPAAT3 KO mice, it should be noted that the decrease in 22:6-PCs in fasting and STZ-injected mouse skeletal muscles might have resulted from a mechanism other than LPAAT3 gene expression. LPAAT3 gene expression was elevated by fasting and STZ injection, which contradicted the decrease in the majority of 22:6-PCs in the mice. In addition, although fasting decreased 22:6-PCs in both the EDL and soleus, LPAAT3 gene expression was altered only in the EDL. Taken together, phospholipid alterations during fasting may not result from the expression of genes-related to phospholipid or fatty acid synthesis and are likely independent of insulin-dependent gene expression regulation machinery.

22:6-PCs decreases and 18:2-PCs increases have been observed in other mouse muscle atrophy models [21, 24], and this suggests that other factors may also be involved in modulating skeletal muscle PC profiles. The cellular 22:6-PC content depends on the availability of 22:6. When mice were fed 18:2 with titrated amounts of 22:6, even a small increase in dietary 22:6 induced a drastic increase in 22:6-phospholipids in the heart [40]. In addition, a dose- and time-dependent increase in 22:6 in skeletal muscle phospholipids was observed with intake of fish oil, in which 22:6 was abundant [41]. These suggest that 22:6 is easily taken up by cellular phospholipids once supplied. In the context of fasting, it is speculated that elongation and desaturation of fatty acids may be prohibited due to the limited supply of NADH or NADPH upon energy depletion. This may lead to a lower availability of cellular 22:6 than in the fed state. In addition, it has been reported that 22:6 is taken up by the liver, loaded into lipoproteins, and delivered to peripheral tissues, while 18:1 is not [42]. It is possible that liver metabolism affected by fasting or insulin depletion may alter the distribution of 22:6 in the liver. Other than these theories, one possible explanation for the compensatory increases in 18:2-PCs in muscles is that 18:2-PCs are pooled in skeletal muscle, possibly via fatty acid transporters or binding proteins, and utilized for phospholipid remodeling. This possible mechanism is supported by a recent study that showed that tissue-specific differences in acyl-chains in cardiolipin depend on how much 18:2-PCs are pooled in each tissue [17]. To our knowledge, neither fatty acid transporters nor binding proteins that show tissue-specificity and/or a preference for specific fatty acids have been identified. Future studies that focus on how cellular phospholipid pools are created will be important for understanding the complex phospholipid diversity. In addition, the precise mechanism(s) that regulate the 18:2- and 22:6-PC balance in skeletal muscle requires further elucidation.

PUFA-containing phospholipid perturbations are associated with the muscle-wasting phenotype [24]. Consistent PC alterations occur in two types of muscle-wasting model mice, namely denervation and mdx (model for Duchenne muscular dystrophy), in which 18:2-PCs increase and 22:6-PCs decrease [24]. The perturbations are rescued in mdx mice that carry truncated dystrophin [24]. Importantly, skeletal muscle structure and functions are normal in rescued mice [43]. Given that fasting also causes a loss of muscle mass, phospholipid perturbations may reflect skeletal muscle mass. Indeed, altered PC profiles upon fasting and STZ-injected statuses correlated with muscle mass (Fig 6B). However, whether the fasting-mediated maintenance of PUFA-containing PCs that is attributed to 18:2-PCs increases affects muscle function remains unclear. Although a large amount of 18:2-PCs replaced the loss of 22:6-PCs in LPAAT3-KO muscles, skeletal muscle mass was not altered (Fig 5B). No significant link was observed between PC profiles and muscle mass in the correlation tests performed with all models (Fig 6A) and in those focused on LPAAT3-KO mice (Fig 6B). These suggest that PC perturbation itself might not be a trigger for muscle-wasting. Even so, this issue should be further investigated by functional analyses using LPAAT3-KO mice combined with exposure to environmental changes or stimuli inducing muscle-wasting. In terms of the proportions of individual fatty acids among the total fatty acids derived from phospholipids, human skeletal muscle contains less 22:6 (2–4%) but more 18:2 (around 30%) [18, 44] compared to mouse skeletal muscle (22:6 21%, 18:2 12%) [21]. This suggests that skeletal muscle functions properly even if phospholipids consist of a relatively high amount of 18:2, although whether phospholipid functions are conserved across species has not yet been determined. Further, n-3 fatty acids, including 22:6, have beneficial effects on skeletal muscle mass and metabolism [45]; while intake of both n-6 and n-3 fatty acids prevents insulin resistance in obese Zucker rats but with different impacts on skeletal muscle mitochondrial metabolism [46]. This implies that it is difficult to determine whether 18:2- or 22:6- PC is more important in skeletal muscle. Finally, why do PUFA-PCs need to be maintained in skeletal muscles? The saturation/unsaturation level of cellular phospholipids is strictly maintained; interfering with the maintenance system results in cytotoxicity [47]. It might be important to maintain a consistent level of PUFA-containing phospholipids in skeletal muscle to maintain proper cellular function. Addressing the involvement of PUFA-containing phospholipids in skeletal muscle functions will expand our understanding of skeletal muscle metabolism and provide new insights into the physiological significance of phospholipids.

## Supporting information

S1 Table Primer sequences for qRT-PCR. GPAT, glycerophosphate acyltransferase; LPAAT, lysophosphatidic acid acyltransferase; AGPAT, acylglycerophosphate acyltransferase; LPGAT, lysophosphatidylglycerol acyltransferase; LCLAT, lysocardiolipin acyltransferase; LPCAT, lysophosphatidylcholine acyltransferase; LPEAT, lysophosphatidylethanolamine acyltransferase; LPIAT, lysophosphatidylinositol acyltransferase; SCD, stearoyl-CoA desaturases; Elovl, elongation of very long chain fatty acids protein; Fads, fatty acid desaturase.
(PDF)

S1 Fig PC alterations upon fasting. Relative amounts of PC species normalized by muscle weight and the peak area of the internal standard. Values represent the mean ± SE (n = 5–7); *P < 0.05 (vs. Fed).
(TIFF)

S2 Fig PC alterations in mice injected with STZ. (A) Relative amounts of PC species normalized by muscle weight and the peak area of the internal standard. Values represent the mean ± SE (n = 5–8); *P < 0.05.
(TIFF)

S3 Fig Proportions of SFA and MUFA-binding PCs. The proportions of PCs to total PC upon fasting (A) and STZ injection (B). Values represent the mean ± SE (n = 5–8); *P < 0.05.
(TIFF)

S4 Fig PC alterations in LPAAT3-KO muscles. Relative amounts of PC species in EDL and soleus normalized by muscle weights and the peak area of the internal standard. Values represent the mean ± SE (n = 3–4); *P < 0.05.
(TIFF)
