# Supplementary material for: Fasting increases 18:2-containing phosphatidylcholines to complement the decrease in 22:6-containing phosphatidylcholines in mouse skeletal muscle
Source: PLoS One. 2021 Jul 26;16(7):e0255178. doi: 10.1371/journal.pone.0255178 (PMC8312970; doi:10.1371/journal.pone.0255178)
Supplement: S1 Table — GPAT, glycerophosphate acyltransferase; LPAAT, lysophosphatidic acid acyltransferase; AGPAT, acylglycerophosphate acyltransferase; LPGAT, lysophosphatidylglycerol acyltransferase; LCLAT, lysocardiolipin acyltransferase; LPCAT, lysophosphatidylcholine acyltransferase; LPEAT, lysophosphatidylethanolamine acyltransferase; LPIAT, lysophosphatidylinositol acyltransferase; SCD, stearoyl-CoA desaturases; Elovl, elongation of very long chain fatty acids protein; Fads, fatty acid desaturase. (PDF) [file pone.0255178.s001.pdf]

S1 Table

	Forward (5'-3')	Reverse (5'-3')
36B4	GGCCCTGCACTCTCGCTTTC	AGGGTCGGCGTGTGTTTCT
GPAT1	AGCAAGTCCTGCGCTATCAT	CTCGTGTGGGTGATTGTGAC
GPAT2	AAGAAAGAGGTACAGCGTATCC	GTGGAGAGCCCTCCTGCACAG
GPAT3	GTACATGCCTCCCATGACTAG	GATCCGTTGCCCACGATCATC
GPAT4	GTGGCAGGACAAGGTCAGAGCTACA	TCCCTCCTGACTCACCAGTTCTTCC
LPAAT1	ACCAGAATGGAGCTGTGGCC	CGCTCCCCCAGGCTTCTTCA
LPAAT2	AAGCGTGAGCTAATGTTCACAGG	TTTTTAAAGGGCAACAGGTCCC
LPAAT3	TGTTCTCAGTGAAGGACCGT	CTTAAGCTCTTGGTTGCCAT
LPAAT4	CAAGATCAATGCCAGACTCTGCT	AAACTTGTGATTGAGGACCACGA
AGPAT5	AGAGGATGCTGCTGTCCCT	AACAAACCACAGGCAGCC
LPGAT1	TCCCAAAGCTGAACCAATAGACA	AATAAAGCGCTGATAGAGCCAGC
LCLAT1	TGGATGTTCCTGTGGAACTGTCT	GGTTCATGGATGGCACAAAAATA
LPCAT1	GTGCACGAGCTGCGACT	GCTGCTCTGGCTCCTTATCA
LPCAT2	GTCCAGCAGACTACGATCAGTG	CTTATTGGATGGGTCAGCTTTTC
LPEAT2	AGAGGGTTAAGTTCTGCCTCCT	CATACAGTCTTCCTCCATCCTGTAA
LPCAT3	TCAGGATACCTGATTTGCTTCCA	GGATGGTCTGTTGCACCAAGTAG
LPCAT4	TTCGGTTTCAGAGGATACGACAA	AATGTCTGGATTGTCGGACTGAA
LPEAT1	CTGAAATGTGTGTGCTATGAGCG	TGGAAGAGAGGAAGTGGTGTCTG
LPIAT1	ATACTGGAACATGACCGTGCAGT	TAGGTAGTAACCAGGGTGGAGGC
Pla2g4e	ATGGTGACAGACTCCTTCGAG	CCTCTGCGTAAAGCTGTGG
Pla2g6	GCCTCGTCAACACCCTCAG	CCTTCACCCGGAATGGGTTC
Pnpla6	CGGGTGCAGAAAACTCCAG	CGCATAATCTTCCGGCCATAGA
Pnpla8	GCAAGAAGTCTTTGTGGGAAACA	CTCACTTTTGTAAGTCCCTTGGG
Pla2g15	GGGTAACCAGTTGGAAGCAAA	TTGTCAATCCAGCAGTCAATGAT

SCD1	CCCCTGCGGATCTTCC	AGGGTCGGCGTGTGTT
SCD2	GAGAAGGGCGGAAAACTGG	ACGAAGCACATCAGCAGGAG
SCD3	AGAAGGGCGGAAAACTGGA	AGCGTGGGCAGGATGAA
SCD4	GGTTCCCTCCTGCAAGGTCTA	ATGATGAGGAAGATGCGTAGGG
Elovl6	TCAGCAAAGCACCCGAAC	AGCGACCATGTCTTTGTAGGAG
Elovl5	GCGCGGGAGAATCCGATATG	GGTTGTTCTTGCGAAGGATGA
Elovl2	GACAGTGCAGGAGAAGGTGATG	GCGTGGTGATAGACATGAAGGA
Fads1	CATTGATCACGACCGGAATG	TGTTGAAGGCTGATTGGTGAA
Fads2	GATGGCTGCAACATGACTATGG	GCTGAGGCACCCTTTAAGTGG
