# Supplementary figures and images for: Fasting increases 18:2-containing phosphatidylcholines to complement the decrease in 22:6-containing phosphatidylcholines in mouse skeletal muscle
Source: PLoS One. 2021 Jul 26;16(7):e0255178. doi: 10.1371/journal.pone.0255178 (PMC8312970; doi:10.1371/journal.pone.0255178)

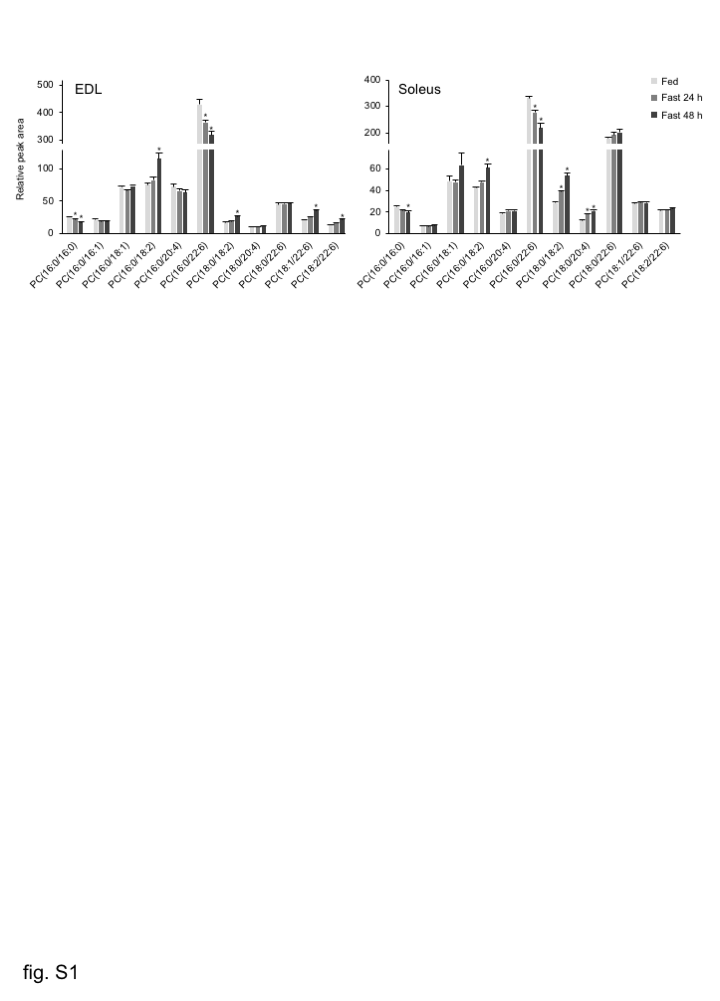

Supplement: S1 Fig — Relative amounts of PC species normalized by muscle weight and the peak area of the internal standard. Values represent the mean ± SE (n = 5–7); *P < 0.05 (vs. Fed). (TIFF) [file pone.0255178.s002.tiff]

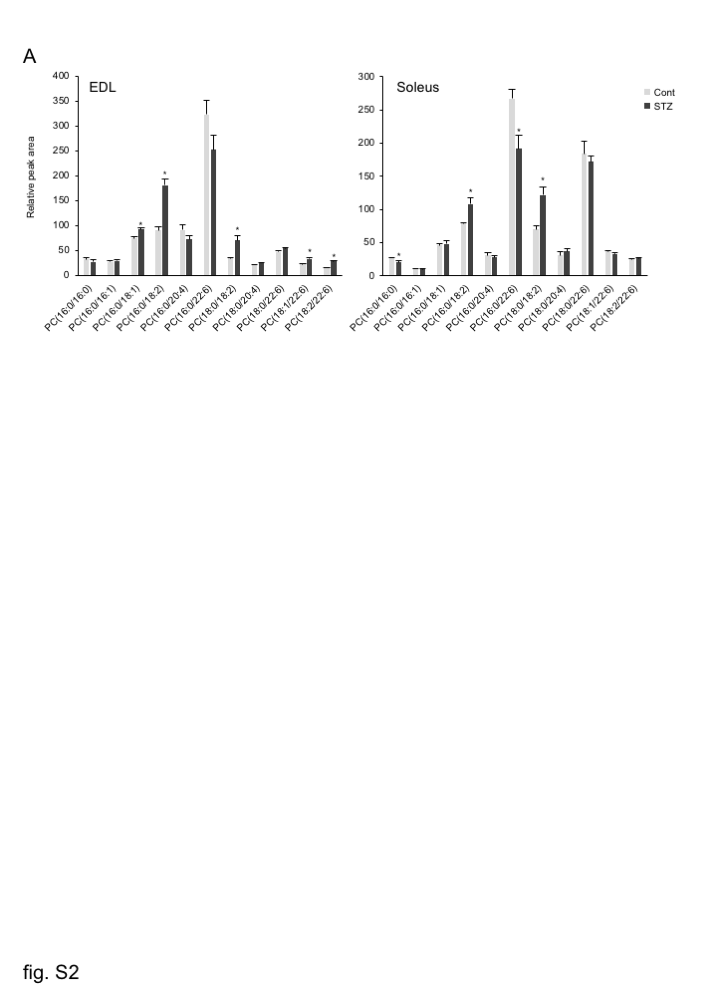

Supplement: S2 Fig — (A) Relative amounts of PC species normalized by muscle weight and the peak area of the internal standard. Values represent the mean ± SE (n = 5–8); *P < 0.05. (TIFF) [file pone.0255178.s003.tiff]

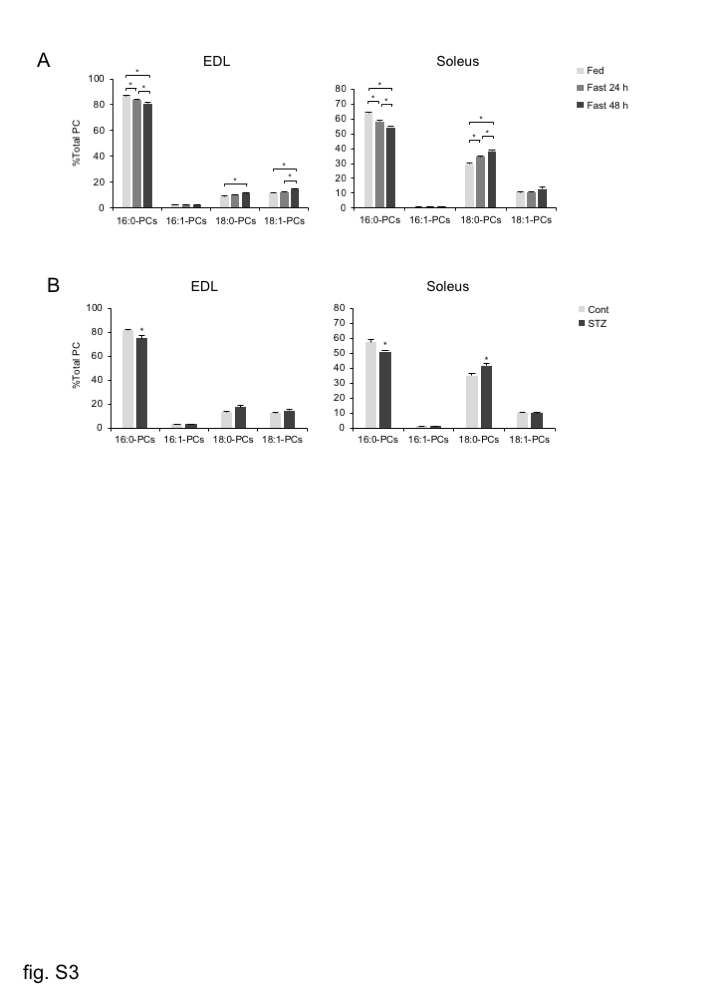

Supplement: S3 Fig — The proportions of PCs to total PC upon fasting (A) and STZ injection (B). Values represent the mean ± SE (n = 5–8); *P < 0.05. (TIFF) [file pone.0255178.s004.tiff]

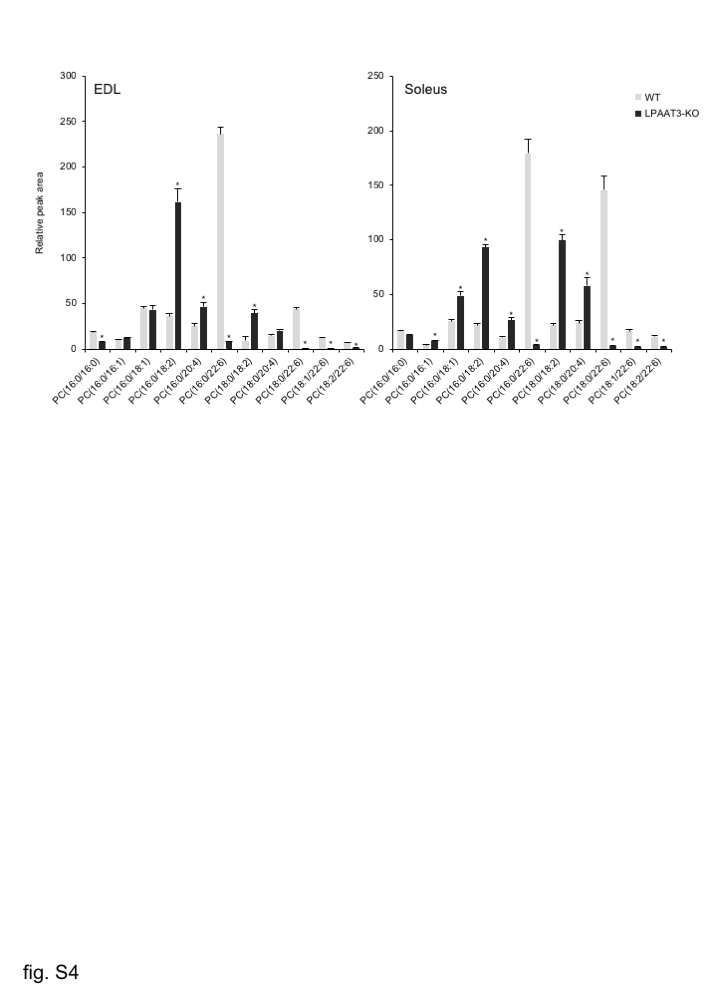

Supplement: S4 Fig — Relative amounts of PC species in EDL and soleus normalized by muscle weights and the peak area of the internal standard. Values represent the mean ± SE (n = 3–4); *P < 0.05. (TIFF) [file pone.0255178.s005.tiff]
